# Combination therapy: the next opportunity and challenge of medicine

**DOI:** 10.1186/1479-5876-9-115

**Published:** 2011-07-21

**Authors:** Paolo A Ascierto, Francesco M Marincola

**Affiliations:** 1 Medical Oncology and Innovative Therapies Unit, Istituto Nazionale per lo Studio e la Cura dei Tumori, Fondazione "G. Pascale", Napoli, Italy; 2 Infectious Disease and Immunogenetics Section (IDIS), Department of Transfusion Medicine, Clinical Center and trans-NIH Center for Human Immunology (CHI) National Institutes of Health, Bethesda, Maryland, 20892, USA

## Abstract

From an historical point of view, combination therapy was the basis for the care of important diseases like infection diseases or cancer. Today the "cocktail drug" of the Highly Active Anti Retroviral Therapy (HAART) has reduced the death for HIV infection changing the outcome of such disease. Moreover, the combination of different strategies changed the course of transplants (both in haematology and surgical transplant). Different diseases with high social impact including cardiovascular, metabolic (obesity, hypercholesterolaemia and diabetes) and autoimmune diseases, have better results with combinations of different drug classes of drugs. After recent successes in the immunotherapy field (Sepuleucel-T, ipilimumab) and the new promising small molecule therapies, cancer should be the next challenge for combination strategies.

## 

In order to accomplish these objectives open discussion and cooperation among companies, academic, and other institutions will be increasingly important. For all of these reasons, we have created a new subsection of the Journal of Translational Medicine.

Why should a Subsection of the Journal of Translational Medicine be dedicated to combination strategies? Because we want to stimulate discussion over this hot topic. In recent years, several compounds have been developed to treat a broad number of diseases targeting a specific mechanism. However, vast majority of common diseases are multi-factorial and cannot realistically be controlled by targeting a single or few pathways.

Tuberculosis treatment is a classic example of combination therapy [1]. After the discovery of Streptomycin in 1944, improvement in the effectiveness of therapy was observed with the addition of Isoniazid, the first oral mycobactericidal drug in 1952 and Rifamycins in 1957. The introduction of Rifampicin in 1970 further improved the effectiveness of the treatment of tuberculosis. As for this example, the main reason for the improved effectiveness of combination therapy is prevention of the emergence of resistance to individual drugs. Moreover, different drugs displaying different pharmacodynamics and/or pharmacokinetics could target subpopulations of mycobacteria independently on metabolic stage or location within the organism.

With the advent of novel immunosuppressive agents allograft rejection is prevented in an increasingly more effective manner with reduced toxicity [2]. Drugs like Cyclosporin A, Tacrolimus (FK506), Voclosporin (ISA247), Sotrastaurin (AEB071), Sirolimus, Everolimus, Mycophenolic acid, Azathioprine, CP-690550, Belatacept (LEA29Y), Alefacept Humanized LFA3-Ig, Basiliximab, Alemtuzumab, Muromonab-CD3, Rituximab, Bortezomib Tripeptide, Eculizumab incrementally reduced the risk of transplant rejection when used in combination. Other important examples of effective combinatorial approaches are represented by Human Immunodeficiency Virus (HIV) and Hepatitis C Virus (HCV) infections. The discovery of several classes of drugs that exert different anti-viral mechanisms dramatically changed the prognosis in these patients. The Highly Active Anti Retroviral Therapy (HAART) changed HIV infection from an incurable disease to a chronic illness. According to Julio Montaner, director of the British Columbia Centre for Excellence in HIV/AIDS, "results show a strong and significant association between increased HAART coverage, reduced community viral load, and decreased number of new HIV diagnoses per year in the population of a Canadian province" [3]. "While waiting for an effective vaccine, experiences such as those reported today should be strongly considered by clinicians, national and international agencies, policy makers, and all parties involved in the development of treatment guidelines, because the population-based dimension of HAART might play an important part in the future control of the HIV epidemic" wrote Franco Maggiolo and Sebastiano Leone [4].

Combination therapy with several drugs is the approach used in several cardiovascular diseases. Acute Coronary Syndrome [5] and Pulmonary Arterial Hypertension [6] are classical examples. Moreover, an abundance of studies demonstrate additive antihypertensive benefit by combining 2 agents of different classes in Essential Hypertension. The extra blood pressure reduction from combining drugs from 2 different classes is approximately 5 times greater than doubling the dose of 1 drug [7]. This is particularly true for thiazide diuretics, which significantly improve blood pressure control when used in combination with most if not all other classes of agents [8]. Often Cardiac Disease are associated with other metabolic diseases like hypercholesterolaemia and diabetes; each of them is further and effectively treated with the association of different compounds [9,10].

Even autoimmune diseases can benefit from combination therapy. Crohn's disease [11] and rheumatoid arthritis (RA) are good examples of such an approach. In fact, recently antitumour necrosis factor (anti-TNF) therapies are now routinely used in the management of rheumatoid arthritis in patients for whom traditional disease-modifying antirheumatic drugs (DMARDs) have failed. A better response has been demonstrated with any of three anti-TNF therapies [etanercept (ETN), infliximab (INF) and adalimumab (ADA)] when used in combination with methotrexate (MTX) than when used as monotherapies (Nixon). Moreover, combining anti-TNF with MTX in combination with one or more other DMARDs [sulfasalazine (SSZ), hydroxychloroquine (HCQ) or both] resulted in better long-term treatment persistence [12].

But this is only the beginning! Treatment of cancer may indeed represent the field where combination strategies surely play a crucial role. In fact, a combination of surgery, radiotherapy, and chemotherapy has been the standard care for the treatment of breast, ovarian, and head and neck cancer. However, in the last decade, the approaches to cancer therapy have changed. In 1998, the approval of trastuzumab for the treatment of breast cancer was a milestone which established a new class of cancer treatment that utilizes immune based mechanisms rather than cytotoxic drugs. In 2001, imatinib was approved for the treatment of chronic myeloid leukemia and its utilization had a dramatic impact on long term survival. Since then, new drugs targeting specific mechanisms have been employed for the treatment of several types of cancer. In some cases promising results had an impact on subsequent approaches (bevacizumab, cetuximab, sorafenib, sutent) whereas other drugs failed to reach the expected results (gefitinib, CpG). Ipilimumab, a further example: a target agent that, through an indirect action on the immune system, induces anti-neoplastic activity against cancers such as melanoma, prostate cancer and NSCLC, has recently obtained Food and Drug Administration (FDA) approval for advanced melanoma treatment [13]. Together with other recent successes in immunotherapy, 2010 became a milestone year for the biological therapy of cancer. In 2010, the first anti-cancer vaccine received FDA approval (Sepuleucel-T for the treatment of prostate cancer) [14] as well as Vemurafinib (PLX4032) or the specific inhibitor of mutated BRAF (V600E) has demonstrated dramatic effectiveness in melanoma patients [15]. Nevertheless, we have new interesting targeted agents (ALK inhibitor, MEK, PI3K and AKT inhibitors, anti-PD1, demethylating agents, oncolytic viral vaccine, etc.) that are ready to be used in clinical trial. Thus, we have been treating cancer attacking one pathways/mechanism at a time disregarding the complexity, redundancy, and compensatory mechanisms typical of this rapidly evolving disease. Thus far, not many examples of combinatorial approaches have been tested; it would be highly desirable to follow the successful example of the treatment of HIV with HAART. A multi-target therapy could combine novel agents with standard chemotherapy or novel agents with different mechanisms or action. Sequential administration of different agents may inhibit cancer cell growth at different check points, while other agents may inhibit neo-angiogenesis, survival of malignant cells or metastases, potentially converting cancer into a chronic disease [16].

Toxicity will surely represent one limitation In fact, if we look at HAART, toxicity from the combination of several drugs should not be overlooked [17]. On the other hand, it is known that the combination of different strategies may amplify the various side effects of every single drugs. However, we must be ready to face such issues if the goal is to turn an incurable lethal disease into a manageable disease.

The challenge will be to test novel combinations particularly for drugs that have not been as yet licensed. Clinical trials are often influenced by the financial pressure aimed at demonstrating product effectiveness for licensing rather than systematically designing trials to evaluate the mechanisms of action of the therapy as well as whether biological end-point are achieved or why treatment might have failed. Such information could provide clues for the design of more sophisticated follow up studies using novel agents. Thus, several drugs tested as single agents risk being withdrawn and becoming unavailable for continue research by early negative clinical results from studies that had not been planned carefully to understand the potential activity of the drug when administered to human beings. It may be important to rescue drugs that "failed" when used as single agents to study their effectiveness in combination with others that have different but potentially synergistic mechanisms of action. Going back to infectious diseases, a good example is the association of the combination between pegylated interferon-alpha and Ribavarin, two drugs that alone demonstrated very minimal benefit, for the treatment of chronic HCV [18].

Testing drugs in combination may be hampered not only by scientific or regulatory hurdles, but also by the difficulty of obtaining the interest and participatin of individual companies. Segregation of products according to market niches and proprietary arguments is limiting the utilization of promising products in combination and their potential application to broad patient populations as licensable combination therapy.

For all the above reasons, we are creating a subsection of the *Journal of Translational Medicine *dedicated to combination strategies as a forum to stimulate discussion between academia, industry and other institutions in order to emphasis the identification of clinical trial strategies utilizing combinations of treatment. Although cancer and infectious diseases have been recently in the front line of the interest for combinatorial approaches, we recognize that other disciplines such as cardiovascular, autoimmune, metabolic and other chronic disorders characterized by complex biology will benefit of drug combination approaches in next future. Therefore, this subsection is now open to submissions relevant to this topic, independently on the target disease.
